# Acknowledgement of manuscript reviewers 2014

**DOI:** 10.1186/s13002-015-0003-9

**Published:** 2015-05-30

**Authors:** Andrea Pieroni

**Affiliations:** University of Gastronomic Sciences, Piazza Vittorio Emanuele 9, 12060 Bra/Pollenzo, Cueno Italy

## Abstract

The editors of *Journal of Ethnobiology and Ethnomedicine* would like to thank all our reviewers who have contributed to the journal in Volume 10 (2014).

**Arshad Abbasi**

Pakistan

**Raziq Abdul**

Pakistan

**Getachew Addis**

Ethiopia

**Selena Ahmed**

United States of America

**Mir Ajab Khan**

Pakistan

**Ulysses P. de Albuquerque**

Brazil

**Romulo Alves**

Brazil

**Eugene Anderson**

United States of America

**Alpina Begossi**

Brazil

**Alessandro Boesi**

Italy

**Jorge Botero**

Brazil

**Colm Bowe**

United Kingdom

**Piero Bruschi**

Italy

**Rainer W Bussmann**

United States of America

**Anja Byg**

United Kingdom

**Alejandro Casas**

Mexico

**Ahmad Cheikhyoussef**

Namibia

**Kevin Cianfaglione**

Italy

**Eraldo Costa Neto**

Brazil

**Gisella S. Cruz-Garcia**

Colombia

**Zora Dajic Stevanovic**

Serbia

**Hugo De Boer**

Norway

**Kathryn Demps**

United States of America

**Loyana Docio**

Brazil

**Fabrizio Frascaroli**

Switzerland

**Orou Gaoue**

United States of America

**Mirutse Giday**

Ethiopia

**Peter Giovannini**

United Kingdom

**Erik Gómez-Baggethun**

Spain

**Louis Evan Grivetti**

United States of America

**Paolo Guidetti**

France

**Karl Hammer**

Germany

**Natalia Hanazaki**

Brazil

**Michael Heinrich**

United Kingdom

**Norma I Hilgert**

Argentina

**Michael Hill**

Ecuador

**Eugene Hunn**

United States of America

**Ashok Jain**

India

**Shujaul Mulk Khan**

Pakistan

**Khasbagan**

China

**Monika Kujawska**

Poland

**Ana Haydee Ladio**

Argentina

**Marco Leonti**

Italy

**Nicolas Lescureux**

Norway

**Rafael Lira**

Mexico

**Chunlin Long**

China

**Reinaldo Lucena**

Brazil

**Lukasz Luczaj**

Poland

**Manuel J. Macia**

Spain

**Alfred Maroyi**

South Africa

**Will Mcclatchey**

United States of America

**Victor Benno Meyer-Rochow**

Germany

**Daniel Moeman**

United States of America

**Zsolt Molnár**

Hungary

**Nemer Narchi**

Mexico

**Mark Nesbitt**

United Kingdom

**Justin Nolan**

United States of America

**Lisa Offringa**

United States of America

**Manuel Pardo-De-Santayana**

Spain

**Berit Smestad Paulsen**

Norway

**Nivaldo Peroni**

Brazil

**Andrea Pieroni**

Italy

**Lisa Leimar Price**

Netherlands

**Rajindra Puri**

United Kingdom

**Cassandra Quave**

United States of America

**Marsha Quinlan**

United States of America

**Subramanyam Ragupathy**

Canada

**Mohammed Rahmatullah**

Bangladesh

**Krishnendu Ray**

United States of America

**Victoria Reyes-Garcia**

Spain

**Nanci Ross**

United States of America

**Juan Manuel Rosso-Londoño**

Colombia

**Nicholas Sadgrove**

Australia

**Valentina Savo**

Canada

**Susan Semple**

Australia

**Krishna Shrestha**

Nepal

**Maria Adele Signorini**

Italy

**Paul Sillitoe**

United Kingdom

**Bradley Simpson**

Australia

**Renata Soukand**

Estonia

**John Richard Stepp**

United States of America

**Ingvar Svanberg**

Sweden

**Tilahun Teklehaymanot**

Ethiopia

**Robert Thornton**

South Africa

**Chusie Trisonthi**

Thailand

**Viktor Ulicsni**

Hungary

**Tinde Van Andel**

Netherlands

**Ina Vandebroek**

United States of America

**James Veteto**

United States of America

**Heike Vibrans**

Mexico

**Robert Voeks**

United States of America

**Christian R. Vogl**

Austria

**Gabriele Volpato**

Netherlands

**Sonia Vougioukalou**

United Kingdom

**Anna Waldstein**

United Kingdom

**Anke Weisheit**

Uganda

**Erdem Yesilada**

Turkey

**Marius Yetein**

Benin

**Haile Yineger**

Ethiopia

**Woldu Zerihun**

Ethiopia

